# latrogenic fracture of humerus – complication of a diagnostic error in a shoulder dislocation: a case report

**DOI:** 10.1186/1752-1947-1-41

**Published:** 2007-07-02

**Authors:** Riaz Ahmad, Shahbaz Ahmed, Michael Bould

**Affiliations:** 1 Department of Trauma and Orthopaedics, Weston General Hospital, Grange Road, Uphill, Weston-Super-Mare, BS23 4TQ, UK

## Abstract

Shoulder dislocation is the commonest dislocation presenting to the emergency department, anterior being more common than posterior. The latter being less common has a tendency of being missed; this is supported by many cases in the literature. Kocher's method is one of the many methods of reducing anterior dislocation; there are many reported complications of employing this method.

To the best of our knowledge we are reporting the first case of an iatrogenic fracture of the proximal humerus, due to the use of Kocher's method of shoulder reduction in a posterior dislocation following a diagnostic error which led to an avoidable difficult surgical intervention. We also discuss the mechanism of the iatrogenic fracture and the measures that can be undertaken to prevent it.

## Background

The shoulder is the most commonly dislocated major joint in the human body comprising up to 45% [1] of dislocations. Posterior shoulder dislocation is rare with an incidence of 1% to 4% [2]. Posterior fracture dislocation is even less common [3]; because of this the diagnosis is often missed.

Kocher's manoeuvre is one of the methods used for reduction of anterior shoulder dislocation. There are many documented complications of the Kochers method of reduction, including injury to the brachial plexus and axillary vessels, avulsion of the rotator cuff [4] and fracture of the humerus during manipulation [5]. Most reported cases of fracture of the humerus following Kocher's manipulation are found in osteoporotic bones [5].

We report a case of fracture in the humerus while using Kocher's method for a posterior dislocation which had been misdiagnosed as an anterior dislocation.

## Case presentation

**Figure 1:**
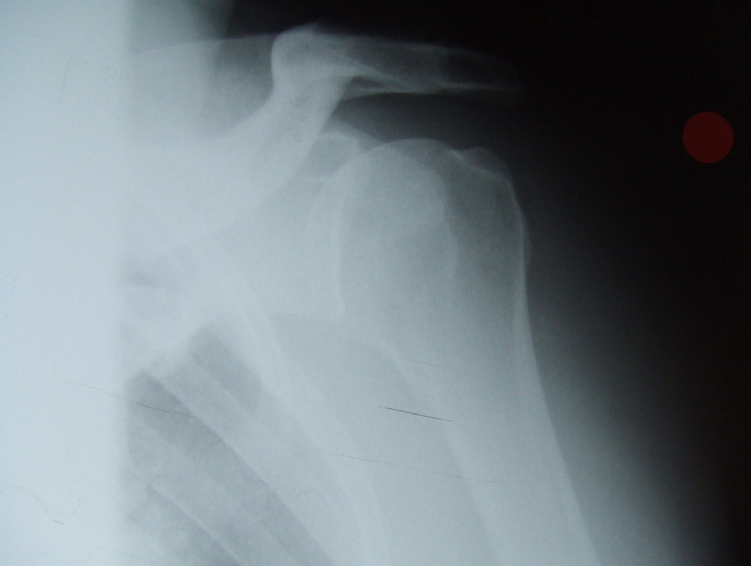
AP view of the shoulder which was diagnosed as anterior dislocation.

**Figure 2:**
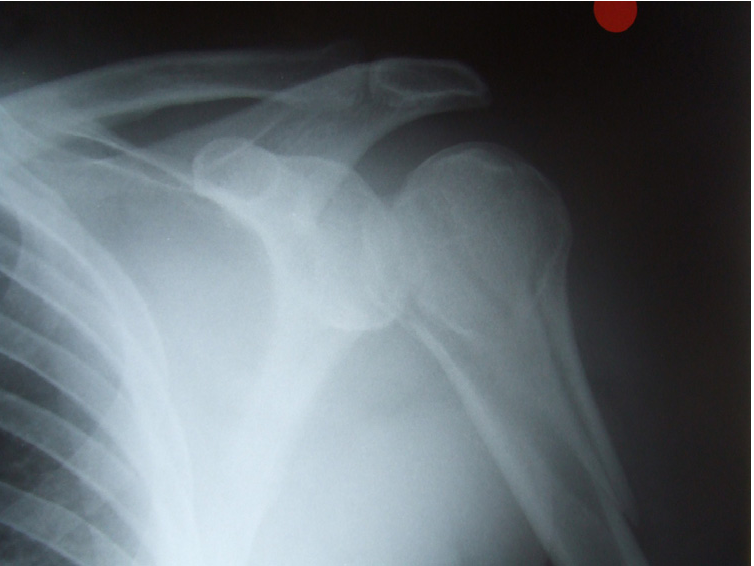
AP view following manipulation showing fracture of the humerus.

A 39 year old, very muscular & athletic male came off a mountain bike at 10 mph landing on an outstretched hand and was unable to use the arm following the injury. On examination in the Accident and Emergency department there was no obvious deformity documented. There was severe pain in the proximal humerus. AP and scapular-Y views of his shoulder were performed and misdiagnosed as showing an anterior shoulder dislocation (Fig. 1). Pre-reduction films did not have some of the classical radiographic features of posterior dislocation such as the empty glenoid or the light bulb sign. These radiographs when reviewed in the orthopaedic department did show a posterior dislocation with a Hill Sach's lesion. The dislocation having been misdiagnosed as anterior, reduction was attempted under sedation [Midazolam] in the A & E using Kocher's method. On attempting the Kochers method a crack was felt and radiographs taken afterwards revealed a comminuted proximal humerus fracture (Fig. 2).

**Figure 3:**
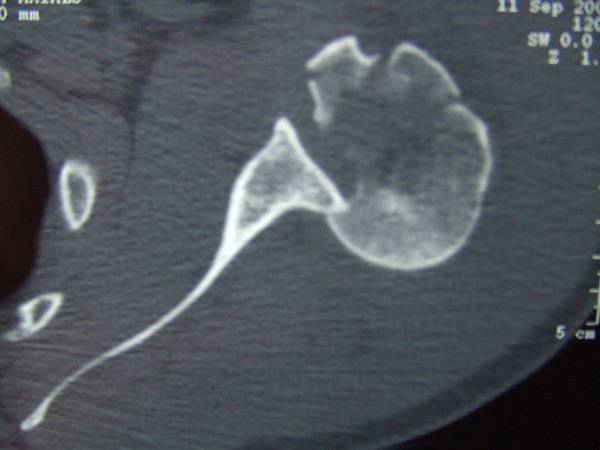
CT scan showing impacted humeral head with Hill-Sachs lesion and fractures of tuberosities.

The patient was referred to orthopaedics for treatment at this point. CT scans revealed a posterior shoulder dislocation, reverse Hill-Sach's lesion, fracture of the lesser and greater tuberosity and spiral fracture extending into the proximal humeral shaft (Fig. 3). The patient was subsequently treated by open reduction and internal fixation. The fracture was found to be a long spiral fracture extending from the reverse Hill Sach's lesion into the proximal humeral shaft which suggested a rotational cause of the fracture. The fracture needed internal fixation.

## Discussion

Posterior shoulder dislocation is uncommon [6], and most frequently occurs following seizures or trauma. High energy trauma causes posterior dislocation when an axial force is applied to the arm with the shoulder in internal rotation, flexion and adduction [6]. Posterior dislocation is often overlooked [7] and early diagnosis is a key for successful treatment. The key to diagnose the dislocation is a high index of suspicion and performing adequate radiological investigations. Despite advances in imaging, posterior shoulder dislocations are frequently missed and diagnosed later. Late diagnosis is a poor prognostic factor in shoulder dislocation [6].

In an isolated posterior shoulder dislocation the impaction fracture of the anterior humeral head (reverse Hill-Sachs) sits on the posterior aspect of the glenoid causing a mechanical block. This mechanical block is disimpacted by clearing the impaction fracture from the glenoid lip by gentle manipulation with the arm being flexed to 90 degrees and adducted [6]. External rotation at this stage will relocate the shoulder, but it should not be attempted before the defect has been fully disengaged, as there is a risk of fracturing the humerus [6]. It is important to consider gentle stretching of the posterior cuff and capsule by maximally internally rotating the arm before attempting to reduce the dislocation.

The Kocher's method described in 1870 is used for the relocation of anterior shoulder dislocations. It states 'the arm, bent at the elbow is pressed against the body. The arm is externally rotated until resistance is experienced. The externally rotated arm is raised in the sagittal plane as far as it will go in a forward direction. Finally, the arm is slowly rotated medially' [8].

We feel that this was a fracture caused by the manipulation and implicate the external rotation part of the Kocher's method. In this case the humeral head impaction fracture was locked on the glenoid and the force of external rotation split the humerus open at the site of impaction. This is supported by the absence of a fracture on the pre reduction radiograph, the feeling of a crack during manipulation, the presence of a spiral fracture on the post manipulation radiograph, the CT findings of impaction of the humeral head and most importantly our peroperative findings of a long spiral fracture suggestive of a rotational force as the culprit.

## Conclusion

This to the best of our knowledge is the first reported case of an iatrogenic proximal humerus fracture following the use of Kocher's method of reduction for a posterior shoulder dislocation. The direction of the glenohumeral dislocation has implications on the management of patient because posterior dislocations are best treated under general anaesthetic as it is very difficult to treat a conscious, sedated patient [6]. Closed reduction of posterior shoulder should be a gentle procedure and if it fails one should proceed to perform an open reduction.

This case reemphasizes the importance of maintaining a high index of suspicion and employing additional radiological investigations in doubtful cases to define the accurate glenohumeral relationship. The additional radiological imaging could be in the form of axillary/apical oblique views or CT scans [6]. Acute medical staff should be aware of this condition and the importance of early referral to avoid a difficult surgical intervention. A correct diagnosis facilitates proper orthopedic treatment. The fracture of the proximal humerus does complicate the treatment of the dislocation [9] and requires fracture stablisation that has attendant risks of non-union, avascular necrosis, post-traumatic arthritis and infection.

## Competing interests

The author(s) declare that they have no competing interests.

## Authors' contributions

RA was involved in the case directly, performed the literature search and drafted the manuscript.

SA was involved in the literature review.

MB contributed to revising the manuscript, improving its intellectual content and highlighting its clinical relevance.

All authors read and approved the final manuscript.
